# Utilization of palliative care resource remains low, consuming potentially avoidable hospital admissions in stage IV non-small cell lung cancer: a community-based retrospective review

**DOI:** 10.1007/s00520-022-07364-0

**Published:** 2022-11-14

**Authors:** Austin M. Meggyesy, Kerrie E. Buehler, Candice L. Wilshire, Shih Ting Chiu, Shu-Ching Chang, Joshua R. Rayburn, Christopher R. Gilbert, Jed A. Gorden

**Affiliations:** 1 Division of Thoracic Surgery and Interventional Pulmonology, Swedish Cancer Institute, 1101 Madison Street, Suite 850, Seattle, WA 98104 USA; 2 Center for Cardiovascular Analytics, Research and Data Science (CARDS), Providence St. Joseph Health, Portland, OR USA

**Keywords:** Non-small-cell lung cancer, Palliative care, Ambulatory care, Emergency service, hospital, Retrospective studies, Health resources, Referral and consultation

## Abstract

**Purpose:**

Early referral of patients with stage IV non-small cell lung cancer (NSCLC) to outpatient palliative care has been shown to increase survival and reduce unnecessary healthcare resource utilization. We aimed to determine outpatient palliative care referral rate and subsequent resource utilization in patients with stage IV NSCLC in a multistate, community-based hospital network and identify rates and reasons for admissions within a local healthcare system of Washington State.

**Methods:**

A retrospective chart review of a multistate hospital network and a local healthcare system. Patients were identified using ICD billing codes. In the multistate network, 2844 patients diagnosed with stage IV NSCLC between January 1, 2013, and March 1, 2018, were reviewed. In the state healthcare system, 283 patients between August 2014 and June 2017 were reviewed.

**Results:**

Referral for outpatient palliative care was low: 8% (217/2844) in the multistate network and 11% (32/283) in the local healthcare system. Early outpatient palliative care (6%, 10/156) was associated with a lower proportion of patients admitted into the intensive care unit in the last 30 days of life compared to no outpatient palliative care (15%, 399/2627; *p* = 0.003). Outpatient palliative care referral was associated with improved overall survival in Kaplan Meier survival analysis. Within the local system, 51% (104/204) of admissions could have been managed in outpatient setting, and of the patients admitted in the last 30 days of life, 59% (87/147) experienced in-hospital deaths.

**Conclusion:**

We identified underutilization of outpatient palliative care services within stage IV NSCLC patients. Many patients with NSCLC experience hospitalization the last month of life and in-hospital death.

**Supplementary Information:**

The online version contains supplementary material available at 10.1007/s00520-022-07364-0.

## Introduction

Palliative care resources are available with the primary goal of easing symptoms associated with advanced disease and facilitating end-of-life care. Lung cancer incidence has steadily decreased; however, it remains the leading cause of cancer-related death (estimated 142,670 deaths in 2019) [1]. In a landmark randomized controlled trial of stage IV non-small cell lung cancer (NSCLC) patients, early referral to outpatient palliative care, defined as within 11 weeks of diagnosis, was associated with increased survival and improved quality of life, as well as a reduction in the unnecessary use of healthcare resources and increased documentation of resuscitation preferences [2]. Following this study, the World Health Organization created the first global resolution on palliative care in 2014, calling for members to implement and strengthen palliative care policies [3]. In addition, the American Society of Clinical Oncology updated clinical practice guidelines to recommend early integration of palliative care into standard oncology care of patients with advanced cancer and the National Quality Forum endorsed metrics to measure quality and aggressiveness of end-of-life care [4, 5].

Prior research has lacked a real world setting to observe outpatient palliative care within a concentrated population, but controlled trials have continued to identify improved overall survival and reduced resource utilization [6, 7]. In addition, studies have attempted to repeat these findings evaluating all cancers in medical systems such as the Veteran Health Administration and within palliative care units in hospitals [8–11].

Our aim was to determine the utilization of outpatient palliative care and the subsequent end-of-life resource utilization in patients with stage IV NSCLC in our multistate hospital network. We additionally evaluated our local healthcare system to identify admissions into the emergency department (ED), hospital, telemetry unit, intermediate care unit, and intensive care unit (ICU) in the last 30 days of life.

## Methods

This study is a retrospective cohort review utilizing data from electronic medical records from centers associated with the Providence Health and Services network. Ethical approval was obtained from the Swedish institutional review board. A query of electronic medical records was made, and patients were included if diagnosed with stage IV NSCLC within Providence Health and Services network between January 1, 2013 and March 31, 2018. Providence Health and Services network spans five states (Alaska, California, Montana, Oregon, and Washington State) encompassing 37 centers, of which 76% (28/37) had dedicated outpatient palliative care services. Patient electronic medical records were queried for, clinical stage, hospital length of stay, ED/ICU admissions, and end-of-life preferences. Outpatient palliative care referrals were also identified, including timing related to diagnosis.

We performed a sub-analysis of patients within our local healthcare system, Swedish Healthcare Network, which resides within the Providence Health and Services network (Supplementary Figure). Swedish Healthcare comprises seven sites all of which possess outpatient palliative care services. Electronic medical records were queried for admissions in the last 30 days of life. Admitting symptoms, imaging studies, treatments, procedures/surgeries, length of stay, in-hospital deaths, and end-of-life wishes were recorded for each visit. Treatments were classified by whether they had the potential for outpatient management, and procedures/surgeries by whether they were considered palliative care.

### Definitions

Early outpatient palliative care referral was defined as occurring within 11 weeks of NSCLC diagnosis, whereas late was > 11 weeks [2]. No outpatient palliative care was defined as having either no palliative care referral or a referral for inpatient palliative care [12]. In our community palliative care outpatient setting, the patients are seen by a palliative care physician specialist.

We utilized three of six endorsed National Quality Forum measures for the “Care of Patient at the End-of-Life” for which we had data available: proportion of patients who died from cancer admitted to the ICU, proportion of patients not admitted to hospice, and proportion admitted to hospice for less than three days [5].

Admissions were categorized as ED, hospital, telemetry unit, intermediate care unit, or ICU. A single overall admission was defined as a summation of consecutive admissions into different departments. Admissions in the last 30 days of life include only those admitted within 30 days or less before death, excluding admissions outside of the last 30 days which roll into the last 30 days. Direct admissions are defined as patients admitted directly from a treatment center, clinic, or for surgery. Lung cancer–related admissions are defined as an admission where symptoms could be attributed directly to lung cancer.

Management with palliative care only is defined as admissions where treatment for NSCLC symptoms could be accomplished with only palliative care. Admissions with potential outpatient management were defined by the patient receiving only interventions which could be administered in an outpatient setting. This excludes any intervention that requires inpatient admission. For example, a patient requiring mild fluid resuscitation and pain management could be managed purely in an outpatient setting and does not necessarily need an inpatient admission.

### Statistical analysis

#### Multistate hospital network

Statistical analysis was performed by the Center for Cardiovascular Analytics, Research and Data Science. Pairwise *p* values were generated based on the following tests: Chi-square test or Fisher exact tests were used for categorical variables including states, end-of-life directives, ED visits, ICU admissions and mortality within 30 days; and Wilcoxon rank-sum tests were used for continuous variables such as age, time from diagnosis to referral, referral to death, length of stay, and continuous covariates. Median survival times were estimated and tested using log-rank test based on survival functions. *P* value comparisons were based on a significance level of 5%. Bonferroni adjustments were made for multiple comparisons resulting in some non-significant *p* values less than 0.05. Overall survival was compared using the Kaplan–Meier method with log-rank test.

Multivariable Cox proportional-hazards modeling was performed to examine for independent effects on the risk of death. Statistical significance was defined as *p* < 0.05. All statistical analyses were performed using R software, version 3.6.0 (R Core Team 2019) and SPSS 24.0 statistical software package (SPSS Inc., Chicago, IL, USA).

#### Local healthcare system

Continuous variables were summarized using median and interquartile ranges. Categorical variables were summarized using counts and percentages. Univariate comparisons were performed using the Chi-squared test for categorical, and Welch’s *t* test or non-parametric Wilcoxon rank-sum tests for continuous covariates, followed by multivariable logistic regression to determine independent factors for whether patients were admitted in the last 30 days of their life or not.

## Results

### Multistate Hospital Network

We identified 3399 patients diagnosed with stage IV NSCLC between January 1, 2013 and March 1, 2018, via an electronic medical record query in our multistate, community-based hospital network. There were 555 exclusions: 494 for unknown death dates and 61 for having inaccessible data within their electronic medical record. Thus, 2844 patients were included in the analysis. Of the 2844 patients, 8% (217/2844) received outpatient palliative care referral, with 72% (156/217) occurring early, although this varied by state (Table 1).

**Table 1:** Multistate hospital network: healthcare resource utilization (*n* = 2844)

	No OPC	Early OPC	Late OPC	*p* values		
	*n* = 2627	*n* = 156	*n* = 61	No OPC versus early OPC	No OPC versus late OPC	Early OPC versus late OPC
OPC per state, *n* (%)						
Alaska	153 (6)	0 (0)	1 (2)	** < **0.001	0.049*	** < **0.001
California	485 (18)	7 (4)	6 (10)			
Montana	154 (6)	4 (3)	4 (7)			
Oregon	648 (25)	106 (68)	24 (39)			
Washington	1,187 (45)	39 (25)	26 (43)			
Age, median (IQR)	70 (62, 78)	70 (62, 77)	68 (60, 78)	0.490	0.192	0.482
ED admissions in last 30 days of life						
Patients with ≥ 1 admission, *n* (%)	1144 (44)	68 (44)	33 (54)	0.992	0.101	0.163
Patients with > 1 admission, *n* (%)	291 (11)	20 (13)	8 (13)	0.766	0.256	0.322
ICU admissions in last 30 days of life						
Patients with admission, *n* (%)	399 (15)	10 (6)	12 (20)	0.003	0.336	0.004
Patients with > 1 admission, *n* (%)	35 (1)	2 (1)	0 (0)	0.008	0.302	0.004
Admissions per patient, median (IQR)	1 (1, 1)	1 (1, 1)	1 (1, 1)	0.223	0.286	0.143
Days in ICU, median (IQR)	3 (2, 7)	5 (3, 8)	2 (1, 3)	0.209	0.065	0.044*
End-of-life wishes						
None, *n* (%)	1879 (72)	104 (67)	38 (62)	0.193	0.115	0.543
Advanced directive and/or power of attorney, *n* (%)	748 (28)	52 (33)	23 (38)			
Diagnosis to OPC, median days (IQR)	-	23 (13, 37)	259 (133, 429)	-	-	** < **0.001
OPC to death, median days (IQR)	-	158 (55, 336)	150 (58, 307)	-	-	0.596
Diagnosis to death, median days (IQR)	108 (100,116)	181 (149, 216)	534 (426, 608)	0.002	** < **0.001	** <** 0.001

*CI*, confidence interval; *OPC*, outpatient palliative care; *IQR*, interquartile range; *ICU*, intensive care unit; *ED*, emergency department

^*^Bonferroni adjustment for multiple comparisons results in *p* value < 0.05 yet not concluded as significant

Overall, 43% (1245/2844) of patients had ≥ 1 ED admission. However, there was no significant difference in the proportion of patients with no outpatient palliative care referral (1144/2627, 44%), early OPC referral (68/156, 44%) or late outpatient palliative care referral (33/61, 54%) (Table 1).

Early outpatient palliative care referrals (10/156, 6%) were associated with less patients admitted to the ICU in the last 30 days of life than patients with a late OPC referral (12/61, 20%; *p* = 0.004), as well as no outpatient palliative care referral (399/2627, 15%; *p* = 0.003). The median number of ICU admissions per patient was 1 (IQR: 1, 1). There was no difference in the ICU length of stay between the groups (Table 1).

**Fig. 1:**
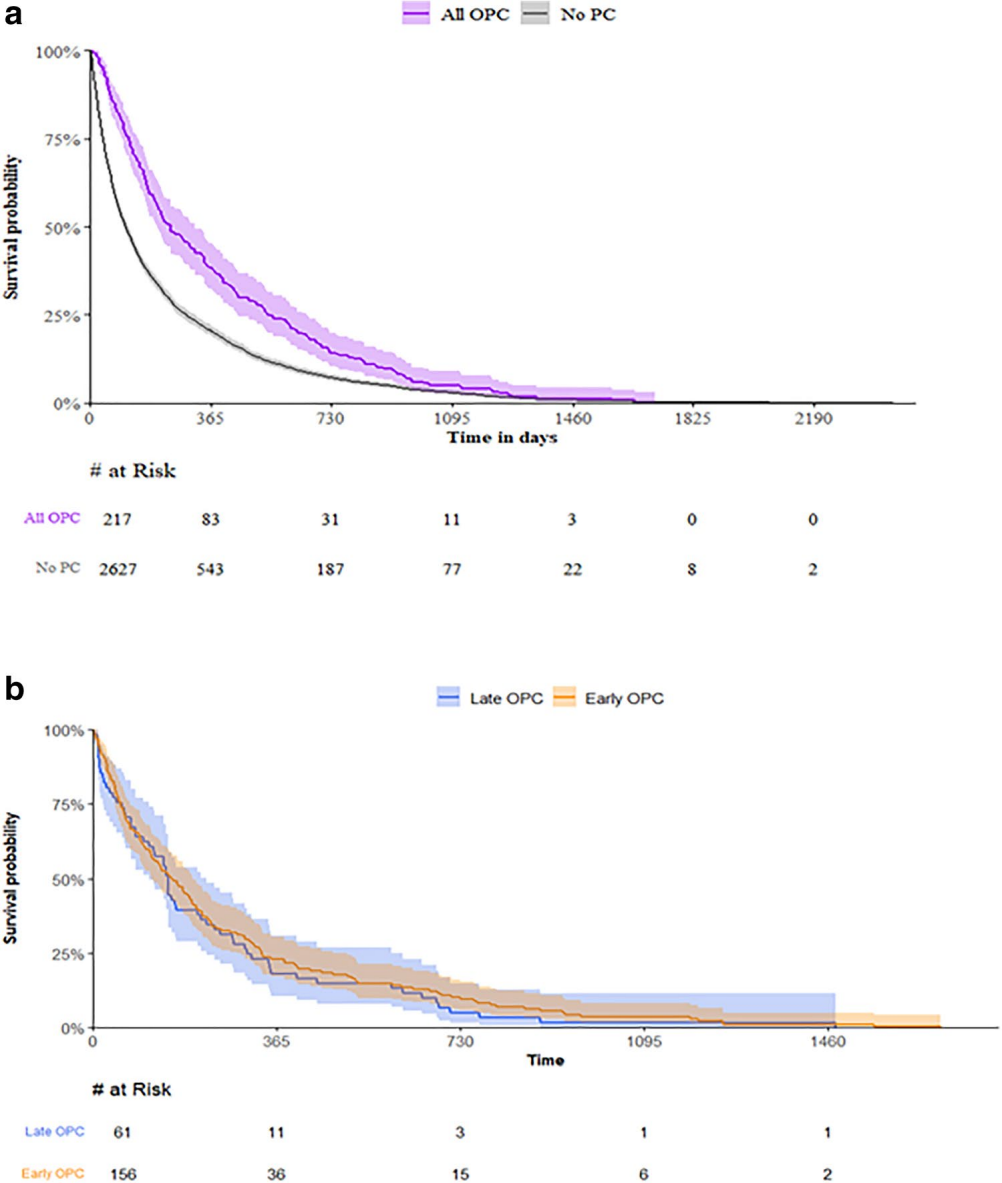
Kaplan–Meier curve comparing. **a** survival from diagnosis to end of follow-up between patients with or without PC treatment, and **b** from PC treatment to end of follow-up between early or late PC treatments

Outpatient palliative care timing had no effect on end-of-life documentation (Table 1). There was no difference in the median days from outpatient palliative care referral to death in early outpatient palliative care (158; IQR: 55, 336) versus late outpatient palliative care referral (150; IQR: 58, 307; *p* = 0.596). There was a significant difference in the median days from diagnosis to death in early outpatient palliative care (181; IQR: 149, 216) compared to late outpatient palliative care referral (534; IQR: 426, 608; *p* < 0.001), as well as to no outpatient palliative care (108; IQR: 100, 116; *p* = 0.002). Kaplan–Meier method analysis estimated patients with both early and late outpatient palliative care as having greater overall survival compared to those with no outpatient palliative care (Fig. 1a). Kaplan Meier analysis demonstrated no difference between early and late outpatient palliative care (Fig. 1b).

No OPC referral was associated with a 3.9 adjusted odds ratio (aOR) for 30-day mortality when compared to early outpatient palliative care (Table 2). The hazard ratio (HR) adjusted by age and state was 1.5 (95% CI: 1.3, 1.8) times higher in patients with no outpatient palliative care compared to all those with outpatient palliative care, and 1.1 (95% CI: 0.8, 1.5) in early outpatient palliative care versus late outpatient palliative care.

**Table 2:** Multistate hospital network: outcomes (*n* = 2844)

Restricted mean survival times		
Time	Differences in days (95% CI)	Ratio (95% CI)
	*No OPC versus all OPC*	
90 days	16 (13, 19)	1.2 (1.2, 1.3)
6 months	40 (32, 47)	1.4 (1.3, 1.4)
1 year	78 (60, 95)	1.5 (1.4, 1.6)
	*Early OPC versus late OPC*	
90 days	2 (− 7, 11)	1.0 (0.9, 1.2)
6 months	3 (− 17, 22)	1.0 (0.9, 1.2)
1 year	8 (− 31, 47)	1.0 (0.8, 1.3)
Odds ratio, 30-day mortality		
	Unadjusted estimates (95% CI)	Adjusted by age estimates (95% CI)
No OPC vs early OPC	4.0 (2.0, 7.9)	3.9 (2.0, 7.8)
Hazard ratios, overall survival		
No OPC versus all OPC	1.5 (1.3, 1.8)	1.5 (1.3, 1.8)
Late OPC versus early OPC	1.1 (0.8, 1.5)	1. 1 (0.8, 1.5)

CI, confidence interval; OPC, outpatient palliative care

### Local healthcare system

We identified 375 patients between August 2014 and June 2017, of which 92 were excluded for unknown death dates. Thus, 283 patients were included in the analysis. Within the local healthcare system, 11% (32/283) received outpatient palliative care. There was no difference in the proportion of patients admitted within the last 30 days of life who received outpatient palliative care (14/32, 44%) compared to those who did not receive OPC (133/251, 53%; *p* = 0.320).

Outpatient palliative care did not impact two of the three National Quality Forum measurements. The proportion of patients who died from cancer and were not admitted into hospice was 41% (13/32) for outpatient palliative care referrals versus 44% (111/251; *p* = 0.850) for no outpatient palliative care referrals. Of those admitted to hospice, 16% (3/19) of those who received an outpatient palliative care referral spent less than 3 days in hospice compared to 9% (13/140; *p* = 0.411) of those without an outpatient palliative care referral. Outpatient palliative care referral was associated with less patients being admitted into the ICU from 17% (43/251) to 3% (1/32; *p* = 0.038).

Overall, 52% (147/283) of patients were admitted in the last 30 days of life to either the ED, hospital, telemetry unit, intermediate care unit or the ICU. Of those patients, 38% (108/283) had only one admission, 9% (25/283) had two, and 5% (14/283) had three or more admissions. The majority (187) of admissions were to the ED (Table 3). Of the ED admissions, 44% (83/187) occurred Monday to Friday 8am to 5 pm, 27% (51/187) were Monday to Friday after hours, and 28% (52/187) were on the weekend.

**Table 3:** Local healthcare system: admissions, treatments, procedures, and deaths (*n* = 147)

	Admissions				
	ED	Hospital	TELE	IMCU	ICU
Admissions, *n*	187	135	20	34	47
Median length of stay, *n* (IQR)	1 (1, 1)	5 (3, 7)	5 (3, 7)	6 (3, 22)	3 (2, 5)
Admission lung cancer related, *n* (%)	165 (88)	129 (96)	18 (90)	34 (100)	41 (87)
Admitting symptoms*, *n* (%)					
Respiratory	88 (47)	54 (40)	12 (60)	23 (68)	27 (57)
Constitutional/other	66 (35)	55 (41)	2 (10)	12 (35)	4 (9)
Neurological	40 (21)	27 (20)	5 (25)	2 (6)	13 (28)
Gastrointestinal	25 (13)	15 (11)	2 (10)	3 (9)	2 (4)
Circulatory/cardiology	19 (10)	19 (14)	7 (35)	75(15)	8 (17)
Median imaging studies per admission, *n* (IQR)	1 (1, 2)	1 (0, 2)	2 (1, 3)	1 (0, 3)	2 (1, 4)
Treatment category*, *n* (%)					
Cardiology/hematology/circulatory	123 (76)	82 (61)	18 (90)	30 (88)	41 (87)
Pain management/comfort care	68 (36)	105 (78)	13 (65)	20 (59)	30 (64)
Respiratory	59 (32)	56 (41)	10 (50)	25 (74)	31 (66)
Infectious disease	55 (29)	56 (41)	9 (45)	24 (71)	32 (68)
Neurological	61 (33)	33 (24)	4 (20)	8 (24)	28 (60)
Gastrointestinal	8 (4)	4 (3)	1 (5)	0 (0)	2 (4)
Procedures/surgery, *n* (%)					
Thoracentesis	3 (2)	17 (13)	1 (5)	3 (9)	5 (11)
Broncho-/colono-/mediastinoscopy	0 (0)	10 (7)	1 (5)	1 (3)	9 (19)
Biopsy	0 (0)	13 (10)	3 (15)	3 (9)	2 (4)
Drain/tube/catheter/suture placement	7 (4)	6 (4)	2 (10)	3 (9)	10 (21)
Surgery	3 (2)	2 (1)	2 (10)	1 (3)	4 (9)
Intubation/ventilation	8 (4)	0 (0)	0 (0)	0 (0)	15 (32)
IVC filters	0 (0)	1 (1)	1 (3)	1 (3)	1 (2)
Only palliative procedures during visit	3 (2)	12 (9)	2 (10)	4 (12)	1 (2)
In-hospital deaths, *n* (% = deaths/admissions)	3 (2)	49 (37)	4 (20)	10 (29)	21 (45)

*ED*, emergency department; *TELE*, telemetry unit; *IMCU*, intermediate care unit; intensive care unit; *IQR*, interquartile range; *IVC*, inferior vena cava. *Multiple admitting symptoms and/or treatments per admission possible

**Fig. 2:**
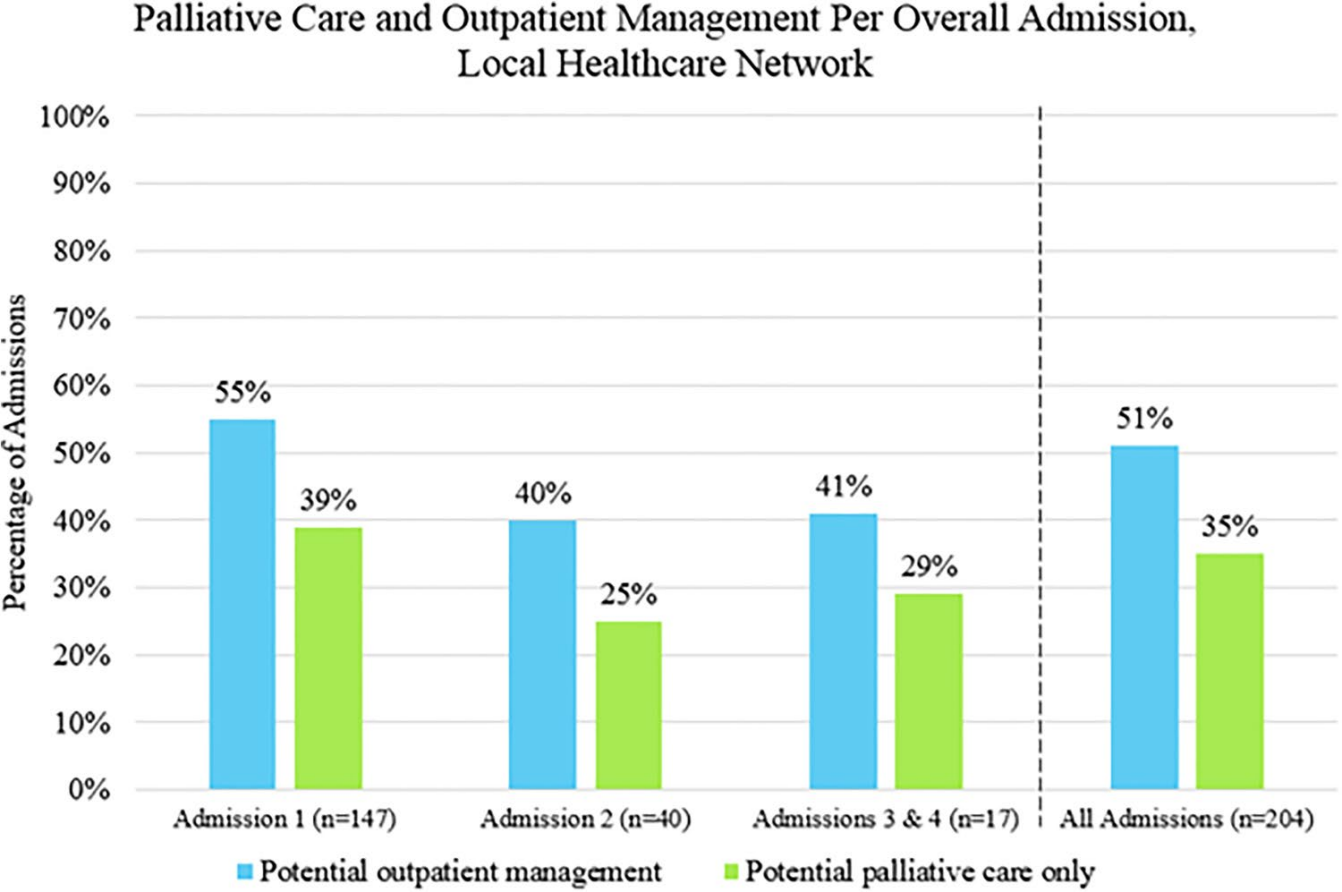
Admissions that could be potentially treated with outpatient management and palliative management. Overall admissions represent consecutive admissions from different departments, for example: patient enters ED then is transferred to HA and then to ICU, this is counted as 1 admission (Table 3)

Admitting symptoms, management and median length of stay for each type of admission is shown in Table 3, and most admissions to each were lung cancer–related. There were 204 overall admissions, with 51% (104/204) having potential for outpatient management and 35% (72/204) having potential for palliative care management only (Fig. 2).

In the last 30 days of life, patients spent a median of 7 (IQR: 4, 14) days in a hospital. Of the 147 patients admitted in the last 30 days of life, 41% (60/147) did not die in the hospital, 67% (40/60) were discharged to outpatient hospice. The remaining 87 patients (59%) admitted within the last 30 days of life died while in the hospital: 3% (3/87) in ED, 56% (49/87) in hospital, 5% (4/87) in telemetry, 11% (10/87) in intermediate care unit, and 24% (21/87) in ICU (Table 3). At the time of death, 63% (92/147) of patients admitted in their last 30 days had a do-not-resuscitate code status.

The odds of admission in the last 30 days of life decreased 16% with a twofold increase in distance lived from hospital (aOR = 0.84; 95% CI = 0.73–0.95) (Table 4). Patients with a code status of do-not-resuscitate were less likely to be admitted (aOR = 0.45; 95% CI = 0.22–0.91).

**Table 4:** Local healthcare system: predictors of admission in the last 30 days of life (*n* = 283)

	Univariate analysis		Multivariate analysis	
	Odds ratio (95% CI)	*p* value	Odds ratio (95% CI)	*p* value
Age at diagnosis	1.01 (0.99, 1.03)	0.343	0.99 (0.97, 1.02)	0.704
Distance from home to hospital (miles)	1 (1.00, 1.00)	0.041	-	-
Distance from home to hospital (miles), log 2	0.85 (0.76, 0.95)	0.006	0.84 (0.73, 0.95)	0.007
Insurance				
Medicare, Medicaid	Reference		Reference	
Private	0.57 (0.3, 1.11)	0.097	0.59 (0.28, 1.24)	0.161
Combination coverage	0.85 (0.47, 1.53)	0.584	1 (0.50, 1.99)	0.994
Tricare/VA/Alaska Native, uninsured, unknown	0.58 (0.14, 2.35)	0.445	0.7 (0.15, 3.15)	0.641
ECOG performance status				
0–2	Reference		Reference	
3–4	1.84 (1.05, 3.22)	0.034	1.44 (0.75, 2.77)	0.272
Type of PC				
None/IPC	Reference		Reference	
OPC	0.69 (0.33, 1.45)	0.327	0.62 (0.26, 1.47)	0.278
Marital status				
Not on file	Reference		Reference	
Married, significant other	1.85 (0.64, 5.33)	0.257	2.9 (0.74, 11.41)	0.128
Divorced, widowed	1.91 (0.61, 5.96)	0.263	1.64 (0.45, 5.94)	0.452
Single	1.87 (0.59, 5.88)	0.286	1.31 (0.36, 4.74)	0.677
Next of kin/surrogate decision maker				
Spouse, significant other	Reference		Reference	
Child, sibling	1.27 (0.77, 2.08)	0.347	1.92 (0.81, 4.57)	0.141
Friend, other	1.73 (0.67, 4.47)	0.256	3.37 (0.84, 13.55)	0.087
Not on file	0.85 (0.22, 3.32)	0.818	0.74 (0.08, 6.73)	0.793
Is decision maker local				
No	Reference		Reference	
Yes	1.22 (0.46, 3.26)	0.695	0.6 (0.18, 2.03)	0.409
Not on file	1.57 (0.35, 7)	0.551	1.17 (0.15, 9.17)	0.884
Advanced directive				
No	Reference		Reference	
Yes	0.88 (0.55, 1.41)	0.600	0.81 (0.46, 1.43)	0.463
Code status prior to last 30 days of life				
Full code	Reference		Reference	
Do-not-resuscitate	0.66 (0.36, 1.2)	0.169	0.45 (0.22, 0.91)	0.026

*CI*, 95% confidence interval; *VA*, veteran’s administration; *ECOG*, eastern cooperative oncology group performance score (0–5); *PC*, palliative care; *IPC*, inpatient palliative care; *OPC*, outpatient palliative care. *Includes patients with data not on file

## Discussion

We identified low utilization of outpatient palliative care in both our multistate hospital network and our local healthcare system. A strength of our study was the unique ability to look at regional palliative care utilization as well as a granular, local review that was able to assess hospital-based resource utilization of palliative care in NSCLC. Our data suggests that outpatient palliative care referrals remain low within the community setting at 8% in our multistate network and 11% in our local healthcare system.

In the small proportion of patients receiving outpatient palliative care referral, we found no difference in time from referral to death; however, we did find a significantly longer survival time associated with late outpatient palliative care referral from diagnosis to death. This finding differs from other reports where the timing of referral has shown to be consequential on symptom intensity and patient survival [2, 10, 11, 13]. It is unclear if this finding is related to the retrospective nature of the study, small numbers, or the community setting compared to prior studies often performed in academic centers. Another explanation could be that palliative care referrals are generated based on performance status, as opposed to objective guidelines [14]. Leaving palliative care referrals up to subjectivity allows for biases and perceptions about palliative care to influence referral patterns and frequency [15, 16]. Current literature suggests a discrepancy around acceptance of outpatient palliative care between providers and patients. Oncologists stated a lack of symptoms reported by patients and concerns that patients may oppose the referral as reasons for not referring to outpatient palliative care early, yet when surveyed, 98% of patients stated they would accept a referral if recommended [17]. It remains unclear how this information may be interpreted by academic versus community oncologists.

An interpretation of our data is that provider reluctance may result in these low referral rates. Further study of automated mechanisms based on national guideline recommendations may improve appropriate outpatient palliative care [18]. Automated suggestions for referrals like a best practice alerts are a potential option, as well as including non-physician providers in the process. Empowering oncology clinic nurses to make referrals may be a valid option. A study of nurses reported they felt involved in the transition to palliative care yet lacked the opportunities to contribute to decision making and referrals [19]. In addition, there has been a proposal to change the name of the service from “palliative care” to “supportive care” to address the perceived barrier that the terminology “palliative care” imparts. While studies report a more positive perception of “supportive care” terminology by medical oncologists and midlevel providers, as well as more inpatient referrals and earlier outpatient referrals; this concept is still controversial and debated among the palliative care community [20–22].

In our local network, outpatient palliative care consultation was not associated with lower admissions of stage IV NSCLC patients. However, chart review suggests that more than a third (35%) of these admissions could potentially be managed in the outpatient setting. We found ED visits were responsible for the greatest number of admissions in the last 30 days of life. Admitted patients spent a median of 7 days in the hospital with the majority suffering subsequent in-hospital death. Previous data of cancer patients suggest an increased likelihood of in-hospital death following unplanned hospital admissions, contrary to patient wishes to spend their final days at home [23]. Outpatient palliative care referral was associated with a reduction in the number of patients being admitted into the ICU in their last 30 days, similar to other studies reporting a lower rate of ICU admissions when referring early palliative care to advanced cancer patients [24].

We identified an association with early outpatient palliative care and decreased ICU admission, but no association to ED visits within the last 30 days. Prior research in small patient populations have shown trends of advanced cancer patients presenting with avoidable ED visits [25–27]. The discordance between these resources remains unclear. Closer review of ED visits noted nearly half (44%), occurred Monday-Friday, between 8am and 5 pm, times when outpatient oncologic resources such as infusion centers are available. Nearly a third of patients (28%) were seen on a weekend suggesting an expansion of outpatient oncologic facilities. In our local healthcare system, more than half of our overall admissions could have been managed with resources available in the outpatient setting. Thirty-five percent required management of symptoms related to NSCLC and are commonly managed with palliative measures alone. Creation of more robust supportive plans with the patient/family and direct pathways to outpatient designated resources for patients with advanced NSCLC may decrease expensive and stressful inpatient experiences and maximize in-home days as opposed to in-patient days. Cancer patients consume a significant amount of health care resources in the last 12 months of life and admissions to the hospital are costly [28, 29]. The emergence and persistence of the COVID-19 epidemic has shined a light on stressed inpatient resources and our need for greater ease of access and integrated clinic referral with outpatient resources for our vulnerable patients with advanced NSCLC.

Due to the retrospective nature of our study design, it remains difficult to elucidate the reason(s) why palliative care does not appear to have the anticipated impact based on prior studies; however, we also theorize that the impact of outpatient palliative care on resource utilization may depend on how the program is embedded in the care of the cancer patients. Differing models may include stand-alone consultations for symptom management versus true integration into treatment teams. We suspect that the act of referral may be insufficient to reduce resource utilization and suggest that measuring outpatient palliative care referral alone may not be the most important quality improvement metric. We are unaware of any current studies evaluating these questions but hypothesize that they may lead to different outcomes.

## Strengths and limitations

Our study is a two-pronged retrospective review examining the use of outpatient palliative care in a real-world setting: this approach allowed us to create a snapshot of a large sample size while providing a more in-depth analysis on a subset. Our study is one of the first to analyze the use of outpatient palliative care within a defined cancer population in a non-academic setting. A limitation of our study is its retrospective design and inherent shortcomings and biases of such design. We assumed all patients within the system were billed thoroughly and had accurate charts. We did not investigate if palliative care consultations were carried out after referral. Billing codes constrained our ability to examine other measurements endorsed by the National Quality Forum. We had access to medical records associated with Providence Health and Services, potentially missing non-network providers of outpatient palliative care. However, many national database queries and compliance are often evaluated in this fashion.

## Conclusion

The utilization of outpatient palliative care in stage IV NSCLC remains low, both within a large multistate network and one of its local healthcare networks. No survival benefit was associated with early versus late outpatient palliative care referral; however, both were associated with a survival benefit over no palliative care. The purported benefit in reduction of resource utilization was found to be weak at best; and most patients within our local healthcare system had in hospital days and death. The largest percentage of hospital resources were used during “normal business hours”, and most admissions were for NSCLC symptoms. Further research needs to examine alternative methods of referral to increase palliative care utilization and greater palliative care integration into care plans to decrease resource utilization considering that many of the admissions have the potential to be managed in the outpatient setting.

## Supplementary Information

Below is the link to the electronic supplementary material.

Supplementary file1 (DOCX 177 KB)

## Data Availability

Data and material can be made available upon request.

## Abbreviations

aOR: Adjusted odds ratio
CI: Confidence interval
ED: Emergency department
HR: Hazard ratio
ICU: Intensive care unit
IQR: Interquartile range
NSCLC: Non-small cell lung cancer
RMST: Restricted mean survival time

## Code availability

Code can be made available upon request.

Approval was granted by the Swedish Medical Center Institutional Review Board (IRB). This is an observational study. The Swedish Medical Center IRB waived the need for ethical approval. This study was conducted retrospectively thus the need to provide individual consent was waived by the Swedish Medical Center IRB.
