# Supplementary material for: Utilization of palliative care resource remains low, consuming potentially avoidable hospital admissions in stage IV non-small cell lung cancer: a community-based retrospective review
Source: Support Care Cancer. 2022 Nov 14;30(12):10117–26. doi: 10.1007/s00520-022-07364-0 (PMC9661463; doi:10.1007/s00520-022-07364-0)
Supplement: Supplementary file 1 — Supplementary file1 (DOCX 177 KB) [file 520_2022_7364_MOESM1_ESM.docx]

**Supplementary Figure 1**


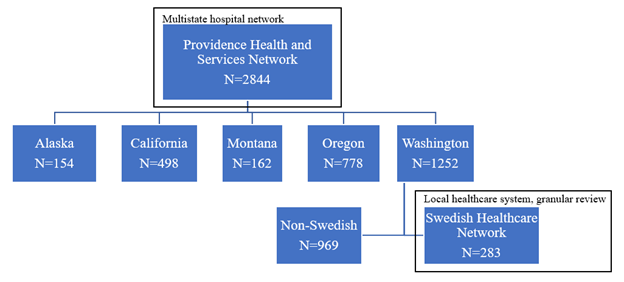


**Supplementary Figure 1.** Consort diagram showing the population sizes within each state; defines the populations of the multistate hospital network and the local healthcare system granular review.
